# *Alchemilla vulgaris* modulates isoproterenol-induced cardiotoxicity: interplay of oxidative stress, inflammation, autophagy, and apoptosis

**DOI:** 10.3389/fphar.2024.1394557

**Published:** 2024-08-07

**Authors:** Nuha Anajirih, Ahmed Abdeen, Ehab S. Taher, Afaf Abdelkader, Hoda A. Abd-Ellatieff, Mahmoud S. Gewaily, Nashwa E. Ahmed, Rasha H. Al-Serwi, Safwa M. Sorour, Heba M. Abdelkareem, Elturabi Ebrahim, Mohamed El-Sherbiny, Florin Imbrea, Ilinca Imbrea, Mahmoud M. Ramadan, Ola A. Habotta

**Affiliations:** ^1^ Department of Medical Emergency Services, College of Health Sciences in Al-Qunfudah, UmmAl-Qura University, Mecca, Saudi Arabia; ^2^ Department of Forensic Medicine and Toxicology, Faculty of Veterinary Medicine, Benha University, Toukh, Egypt; ^3^ Department of Basic Medical and Dental Sciences, Faculty of Dentistry, Zarqa University, Zarqa, Jordan; ^4^ Department of Forensic Medicine and Clinical Toxicology, Faculty of Medicine, Benha University, Benha, Egypt; ^5^ Department of Pathology, Faculty of Veterinary Medicine, Damanhour University, Damanhour, Egypt; ^6^ Department of Anatomy and Embryology, Faculty of Veterinary Medicine, Kafrelsheikh University, Kafrelsheikh, Egypt; ^7^ Department of Medical Biochemistry and Molecular Biology, Faculty of Medicine, Benha University, Benha, Egypt; ^8^ Department of Basic Dental Sciences, College of Dentistry, Princess Nourah bint Abdulrahman University, Riyadh, Saudi Arabia; ^9^ Department of Pharmacology, Faculty of Medicine, Benha University, Benha, Egypt; ^10^ Department of Medical Biochemistry, Molecular Biology and Physiology, Faculty of Medicine, Mutah University, Mutah, Jordan; ^11^ Medical‐Surgical Nursing Department, College of Applied Medical Sciences, Prince Sattam bin Abdulaziz University, Al-Kharj, Saudi Arabia; ^12^ Department of Basic Medical Sciences, College of Medicine, AlMaarefa University, Riyadh, Saudi Arabia; ^13^ Department of Agricultural Technologies, Faculty of Agriculture, University of Life Sciences “King Mihai I” From Timisoara, Timisoara, Romania; ^14^ Department of Forestry, Faculty of Engineering and Applied Technologies, University of Life Sciences “King Mihai I” From Timisoara, Timisoara, Romania; ^15^ Department of Clinical Sciences, College of Medicine, University of Sharjah, Sharjah, United Arab Emirates; ^16^ Department of Cardiology, Faculty of Medicine, Mansoura University, Mansoura, Egypt; ^17^ Department of Forensic Medicine and Toxicology, Faculty of Veterinary Medicine, Mansoura University, Mansoura, Egypt

**Keywords:** *Alchemilla vulgaris*, isoproterenol, myocardial injury, oxidative stress, inflammatory cytokines, HMBG1/RAGE pathway

## Abstract

**Introduction:** Isoproterenol (ISO) is regarded as an adrenergic non-selective β agonist. It regulates myocardial contractility and may cause damage to cardiac tissues. Alchemilla vulgaris (AV) is an herbal plant that has garnered considerable attention due to its anti-inflammatory and antioxidant bioactive components. The present investigation assessed the cardioprotective potential of AV towards ISO-induced myocardial damage.

**Methods:** Four groups of mice were utilized: control that received saline, an ISO group (85 mg/kg, S.C.), ISO + AV100, and ISO + AV200 groups (mice received 100 or 200 mg/kg AV orally along with ISO).

**Results and discussion:** ISO induced notable cardiac damage demonstrated by clear histopathological disruption and alterations in biochemical parameters. Intriguingly, AV treatment mitigates ISO provoked oxidative stress elucidated by a substantial enhancement in superoxide dismutase (SOD) and catalase (CAT) activities and reduced glutathione (GSH) content, as well as a considerable reduction in malondialdehyde (MDA) concentrations. In addition, notable downregulation of inflammatory biomarkers (IL-1β, TNF-α, and RAGE) and the NF-κB/p65 pathway was observed in ISO-exposed animals following AV treatment. Furthermore, the pro-apoptotic marker Bax was downregulated together with autophagy markers Beclin1 and LC3 with in ISO-exposed animals when treated with AV. Pre-treatment with AV significantly alleviated ISO-induced cardiac damage in a dose related manner, possibly due to their antioxidant and anti-inflammatory properties. Interestingly, when AV was given at higher doses, a remarkable restoration of ISO-induced cardiac injury was revealed.

## 1 Introduction

Isoproterenol (isoprenaline hydrochloride, ISO; C_11_H_17_NO_3_) is a synthetic non-selective β-adrenergic receptor agonist, which is the iso-propylamine analog of adrenaline (Song et al., 2020; Pandi et al., 2022). It stimulates heart rate and myocardial contractility and is commonly used to treat bradycardia, heart block, cardiac arrest, and occasionally asthma (Timercan et al., 2019; Asiwe et al., 2023). Cardiotoxicity is one of the most common adverse effects of ISO administration (Pandi et al., 2022). The underlying mechanism of ISO-triggered cardiac damage is complicated and multifactorial, with oxygen-derived free radicals and oxidative stress presumed to be critical mechanisms implicated in ISO cardiotoxicity (Obeidat et al., 2022; Asiwe et al., 2023). ISO generates quinone, which is converted to superoxide anions (O_2_^–^) and hydrogen peroxides (H_2_O_2_) by molecular oxygen (Othman et al., 2017). Overproduction of reactive oxygen species (ROS) causes cellular dysfunction, protein and lipid degradation, and DNA damage, resulting in irreparable cell damage and is associated with the pathogenesis of most cardiovascular conditions (Tsutsui et al., 2011). Although treatment using modern medicine is effective, it is associated with more adverse effects. Therefore, exploring natural products as an innovative alternative for the management of cardiac ailments has recently gained great attention (Abdel-hady et al., 2021; Li et al., 2022). Plant-based drugs are affordable and have fewer side effects (Song et al., 2020). Previous research have demonstrated that herbal remedies or their active compounds can mitigate ISO-induced cardiotoxicity such as *Sanguisorba minor* (Hosseini et al., 2023), *Nerium oleander* Linn (Gayathri et al., 2011), *Fumaria indica* (Sajid et al., 2022), and *Esculetin* (Pullaiah et al., 2021).

*Alchemilla vulgaris* (AV), or “lady’s mantle,” or “lion’s foot,” or “bear’s foot” as known, is an herbaceous perennial plant that corresponds to the Rosaceae family (Vlaisavljević et al., 2019). This herbal plant is quite effective against many gynecological diseases and reproductive problems (Jakimiuk and Tomczyk, 2024). It also is used to treat skin and digestive disorders (Boroja et al., 2018). According to the European Pharmacopoeia 6.0, AV has been designated a medicinal plant with diverse pharmacodynamic activities (Beck, 2008). AV contains a wide range of phytochemical compounds, including tannins (catechin, gallic acid, and ellagic acid), flavonoids (quercetin, luteolin, and proanthocyanidins), phenolic acids, and terpenes, as well as fatty acids and their esters including antibacterial, antioxidant, anti-inflammatory and antifungal properties (Vlaisavljević et al., 2019; Kovač et al., 2022). Several reports have documented the potential of AV against oxidative damage associated with different toxicants, such as cisplatin-induced hepatorenal and testicular injury (Jurić et al., 2020), zinc sulphate-induced reproductive injury (Mohammed and Ali, 2019), and carbon tetrachloride-induced hepatorenal damage (El-Hadidy et al., 2018).

Notwithstanding the considerable research that has been carried out to explain the beneficial effects of AV, there are no published reports investigating the efficacy of AV against myocardial injury. Consequently, this study evaluated the cardioprotective impacts of AV extract on cardiotoxicity induced by ISO injection in albino mice. Cardiac enzyme biomarkers, oxidative state, inflammation-related gene expression levels, apoptotic signaling pathways, and myocardial histoarchitecture were evaluated.

## 2 Materials and methods

### 2.1 Molecular docking assessment

#### 2.1.1 Ligand preparation

Using SDF format, the three-dimensional (3D) structures of ISO were retrieved from the PubChem database (https://pubchem.ncbi.nlm.nih.gov/). Moreover, the 3D structures of AV’s bioactive compounds were retrieved from the LOTUS (https://lotus.naturalproducts.net/) database.

#### 2.1.2 Protein preparation

The 3D structures of mouse glutathione synthetase, CAT, SOD1, SOD2, SOD3, Bcl-2, TNF-α, IL-1β, NF-κB1, Bax, RAGE, and HMBG1 were retrieved from UniProt database (https://www.uniprot.org/). Target protein energy minimization was prepared for docking using UCSF Chimera software.

#### 2.1.3 Visualization and docking interactions

Protein-ligand interactions and docking were carried out using InstaDock (Mohammad et al., 2021), while their visualization was done using BIOVIA discovery visualization 2024 Client software.

### 2.2 Preparation of *Alchemilla vulgaris* extract

The plant material of AV (aerial parts and roots) was procured from a local market in Mansoura, Egypt. Taxonomic identification was provided by the Department of Botany, Faculty of Science, Mansoura University, Egypt. The dried plant material was ground into powder. Each 10 g of dried plant material was soaked and mixed with 100 mL of 60°C distilled water before ultrasound treatment (Ultrasonic Cleaner USC-500 TH, VWR, Darmstadt, Germany) for 1 h at room temperature. Before being used, the plant extracts were filtered. Finally, the AV extract was phytochemically analyzed using HPLC as described in the Supplementary Material and Supplementary Table S1.

### 2.3 Animal study and protocol endorsement

This study was approved by the Institutional Animal Care and Use Committee of the Faculty of Veterinary Medicine, Mansoura University [Approval no. MU-ACUC (VM.R.23.06.69)]. Also, the experimental protocol is in agreement with ARRIVE 2.0 guidelines for animal experiments.

Male albino mice weighing 15–20 g and aged 5–6 weeks old, were procured from the animal unit at the Faculty of Pharmacy, Mansoura University, Egypt. Prior to the experiment, mice were kept in standard housing for 2 weeks under appropriate environmental conditions (21°C ± 2°C, 55% ± 5% humidity, and 12/12 h light/dark cycle). All mice received a standard diet along with unlimited access to water.

After 2 weeks of acclimation, mice were evenly divided into four groups of five mice each. Control group (CTL) mice were given normal saline; ISO group mice were injected subcutaneously (S.C) with ISO (Sigma-Aldrich, MO, United States; 85 mg/kg bw) (Meeran et al., 2021); ISO + AV100 group and ISO + AV200 group mice received the same dose of ISO and 100 or 200 mg/kg of AV extract orally (Özbilgin et al., 2019). Mice were pretreated with AV for seven consecutive days and ISO was given on the 8th and 9th days.

On the 10th day, the experiment was terminated. All mice were euthanized with an intraperitoneal pentobarbital injection (45 mg/kg). Then, blood was withdrawn from the orbital sinus, centrifuged for 10 min at 3,000 ×g and sera collected and stored at −20°C for further biochemical assessment. The heart was rapidly removed and washed with cold physiological saline to remove any blood clots and cut into three pieces. One portion was preserved in buffered formalin (10%) for histopathology. The second fresh piece of tissue from each heart was used to isolate RNA for gene expression analysis and preserved at‒80°C. The remaining fresh tissue piece was kept at −20°C for subsequent oxidative cascade marker investigation.

### 2.4 Assay of cardiac serum markers

Creatine kinase-myoglobin binding (CK-MB) and lactate dehydrogenase (LDH) activities were estimated utilizing colorimetric kits acquired from Human (Wiesbaden, Germany).

### 2.5 Oxidative biomarkers assessment

Oxidative biomarkers, including malondialdehyde (MDA; a lipid peroxidation marker), and reduced glutathione (GSH), were assessed at 532 nm, and at 412 nm, respectively. In addition, antioxidant enzymes as superoxide dismutase (SOD) and catalase (CAT) were measured at 560 nm and 240 nm in cardiac homogenates according to the manufacturer’s instructions (Laboratory Biodiagnostics, Cairo, Egypt).

### 2.6 Assessment of cardiac inflammatory biomarkers

The assessment of inflammation in cardiac tissue homogenates was completed using commercially available ELISA kits for interleukin-1β (IL-1β; Catalog No.; MBS763059, MyBioSource, CA, United States) and tumor necrosis factor-alpha (TNF-α; Catalog No.; LS-F5192, LifeSpan Biosciences Inc., MA, United States) in accordance with the manufacturer’s instructions.

### 2.7 RNA isolation and qRT-PCR

Total RNA was extracted from cardiac tissue samples using TRIzol reagent. cDNA was synthesized utilizing Revert Aid™ H Minus Reverse Transcriptase (Fermentas, Thermo Fisher Scientific Inc., Canada) following the manufacturer’s instructions. PCR was performed with a Quanti Fast SYBR Green RT-PCR kit (Qiagen, Hilden, Germany). All reactions were carried out in duplicate using the ViiA™ 7 System (Thermo Fisher Scientific, CA, United States). The PCR cycling procedures were completed based on the protocols published by Habotta et al. (2023). The ΔΔCt approach was employed to ascertain the relative gene expression levels among the various groups. β-actin was used as the housekeeping gene. The primer sequences (Jena Bioscience, Jena, Germany) used to determine the gene expression levels of B-cell lymphoma protein 2 (Bcl-2), Bcl-2-associated X (Bax), Beclin1, a receptor for advanced glycation end products (RAGE), and NF-κB/p65 are provided in Supplementary Table S2.

### 2.8 Histoarchitectural assessment

The formalin-fixed cardiac tissue samples were dehydrated in a graded alcohol series. Then, the tissues were cleared with xylene prior to paraffin embedding. The tissue blocks were cut into 5 µm thick sections mounted on glass microscope slides, deparaffinized, stained with H&E for histological evaluation, and scanned using a digital camera-integrated imaging system (DM300, Leica, Germany) (Banerjee et al., 2002). Microscopic lesions were graded as “0”, no pathological lesions detected; “1” mild, lesions affecting <10% of heart tissue; “2” moderate, lesions affecting 10%–50% of heart tissues; and “3” severe, lesions affecting >50% of heart tissues (Rizzardi et al., 2012).

### 2.9 Immunohistochemistry analysis

Immunostaining was carried out utilizing the avidin-biotin-peroxidase complex procedure. Briefly, paraffin slices were sequentially deparaffinized and dehydrated using a graded ethanol series. Antigens were retrieved using heat-induced epitope retrieval for 30 min cooled to room temperature for 10 min. Subsequently, cardiac endogenous peroxidases were inhibited for 5 min using a 3% H_2_O_2_ solution. This was followed by three 5-minute washings in phosphate-buffered saline (PBS). The sections were incubated at 4°C overnight with rabbit polyclonal antibodies for high mobility group box 1 (HMGB1; Wuhan Servicebio Technology, Wuhan, Hubei, China, 1:1,000 dilution) and microtubule-associated protein light chain 3 (LC3 I, 1:100 dilution). Then the sections were incubated for 24 h at 4°C with rabbit polyclonal secondary antibodies. The sections were rinsed with PBS, biotinylated goat anti-rabbit IgG was added, and the sections were incubated for 45 min at 30°C with streptavidin-peroxidase complexes. The peroxidase activity was measured using 3,3′-diaminobenzidine. Images were captured with a Nikon Eclipse E200-LED at an original magnification of ×400. Positive-stained regions for HMGB1 or LC3 were evaluated using ImageJ software (version 1.53 t; Wayne Rasband and contributors, NIH, United States). A quantitative scoring system for the immunostained proteins (H-Score) that utilized “Area Percentage” was carried out. Percent Area for the HMGB1 and LC3 stained sections was determined using the Nikon Eclipse E200-LED image analyzer computer system (United States). The image analyzer included a Nikon research microscope, color monitor, color video camera, and a computer hard disc linked to the microscope. The system was controlled using ImageJ software (version 1.53 t; Wayne Rasband and contributors, National Institute of Health, United States). H-Score analysis software was used to record and identify the staining intensity and positive rate (%) of each intensity category for the target cells using the following scale: negative (score; 0), weak, mild (score; 1), moderate (score; 2) and strong (score; 3).

### 2.10 Statistical analyses

Means ± SEM were used to represent the results. A one-way ANOVA was employed to assess the results. Duncan’s test was used as a *post hoc* test to compare the significance among the groups, and *P*-values less than 0.05 were deemed statistically significant. R version 4.0.2 of RStudio was utilized to analyze and visualize the data.

## 3 Results

### 3.1 Molecular docking

**FIGURE 1:**
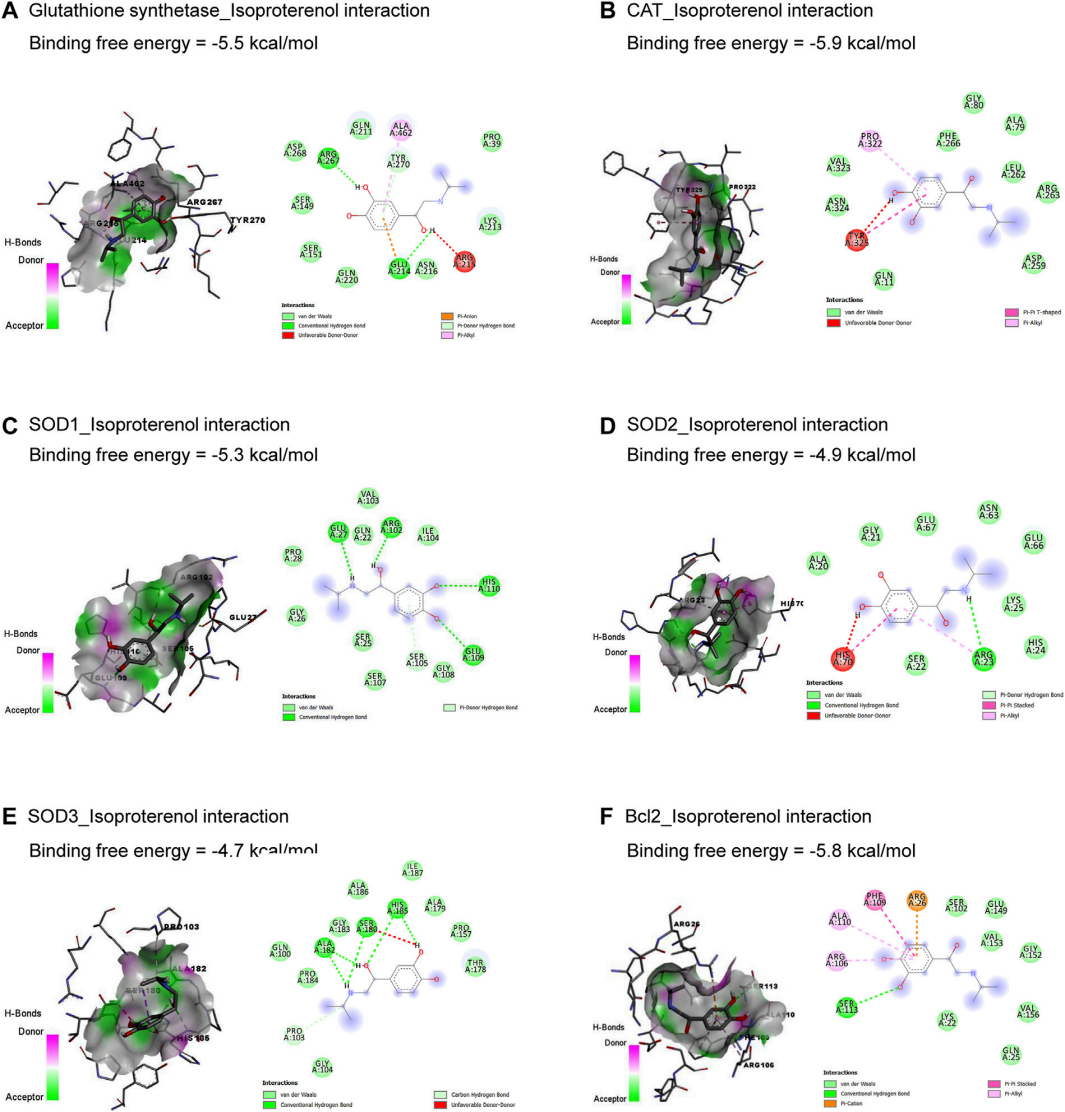
Molecular docking interactions of isoproterenol against glutathione synthetase **(A)**, CAT **(B)**, SOD1 **(C)**, SOD2 **(D)**, SOD3 **(E)**, and Bcl-2 **(F)**. CAT, catalase; Bcl-2, B-cell lymphoma protein 2; SOD, superoxide dismutase.

The molecular interactions of ISO with glutathione synthetase, CAT, SOD1, SOD2, SOD3, and Bcl-2 are represented in Figure 1. ISO interacted with the binding sites of glutathione synthetase (Figure 1A), CAT (Figure 1B), SOD1 (Figure 1C), SOD2 (Figure 1D), SOD3 (Figure 1E), and Bcl-2 (Figure 1F) by binding free energy of −5.5, −5.9, −5.3, −4.9, −4.7, and −5.8 kcal/mol, respectively.

**FIGURE 2:**
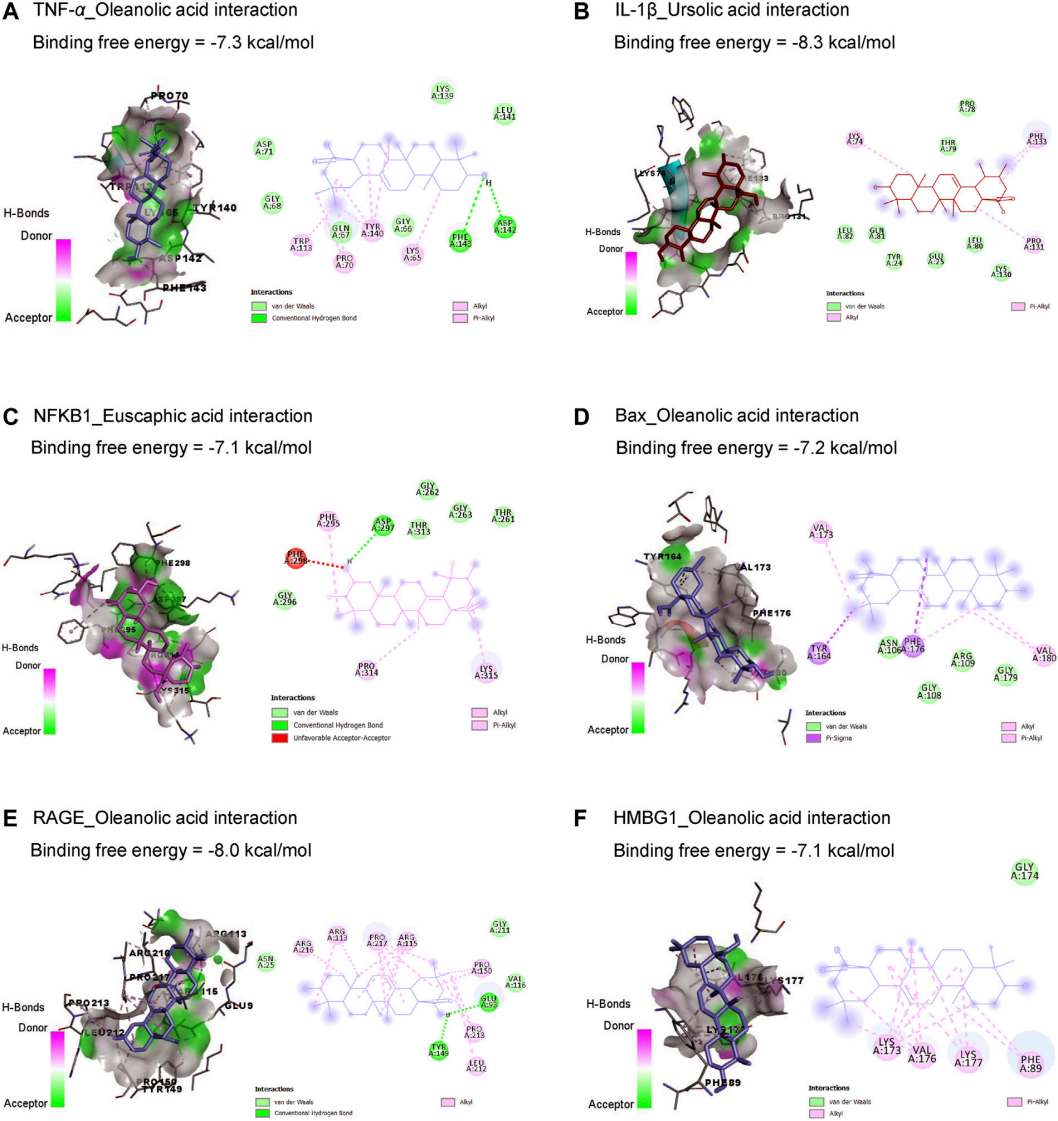
Molecular docking interactions of *Alchemilla* vulgaris’s bioactive compounds with TNF-α **(A)**, IL-1β **(B)**, NF-κB1 **(C)**, Bax **(D)**, RAGE **(E)**, and HMBG1 **(F)**. Bax, Bcl-2-associated X; HMGB1, high mobility group box 1 protein; IL-1β, interleukin-1β; NF-κB, nuclear factor Kappa-B; TNF-α, tumor necrosis factor-α.

Results in Table 1 and Figure 2 explored the molecular docking interaction of AV’s bioactive compounds with TNF-α, IL-1β, NF-κB1, Bax, RAGE, and HMBG1. Oleanolic acid interacted with the binding sites of TNF-α (Figure 2A), Bax (Figure 2D), RAGE (Figure 2E), and HMBG1 (Figure 2F) by binding free energy of −7.3, −7.2, −8.0, and −7.1 kcal/mol, respectively. Another compound, ursolic acid, was bound to the binding site of IL-1β by binding free energy of −8.3 kcal/mol (Figure 2B). In addition, euscaphic acid interacted with NF-κB1’s binding site with −7.1 kcal/mol binding free energy (Figure 2C).

**TABLE 1:** Molecular docking interaction of *Alchemilla* vulgaris’s bioactive compounds against TNF-α, IL-1β, NF-κB1, Bax, RAGE, and HMBG1.

Ligands	Binding free energy (kcal/mol)					
	TNF-α	IL-1β	NF-κB1	Bax	RAGE	HMBG1
Corosolic acid	−7.0	−8.0	−6.6	−7.0	−7.6	−7.1
Euscaphic acid	−7.1	−7.8	−7.1	−7.0	−7.5	−6.8
Oleanolic acid	−7.3	−8.0	−6.8	−7.2	−8.0	−7.1
Quercetin	−6.4	−6.8	−6.0	−6.2	−6.5	−6.3
Tormentic acid	−6.7	−7.8	−7.0	−7.0	−7.4	−6.9
Ursolic acid	−7.2	−8.3	−6.7	−7.0	−7.6	−7.1

### 3.2 Cardiac function parameters

**FIGURE 3:**
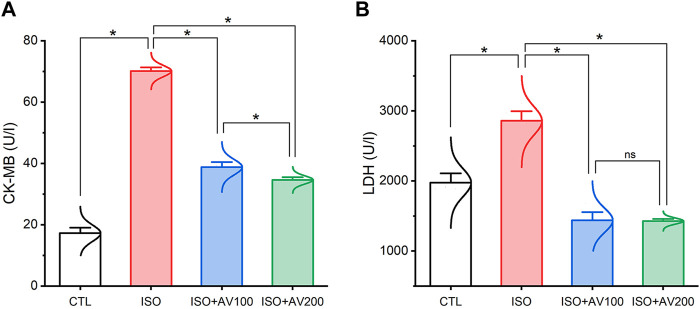
Bar plots of cardiac function parameters of ISO-exposed mice with AV supplementation. **(A)** CK-MB, creatine kinase-myoglobin binding; **(B)** LDH, lactate dehydrogenase. Values are expressed as means ± SEM (*p < 0.05). Colored spikes indicate the data distribution. AV, *Alchemilla vulgaris*; CTL, control; and ISO, isoproterenol.

ISO-induced cardiac injury was characterized by a notable increase in CK-MB and LDH compared to the normal control group. On the contrary, treatment with AV extract at either a high (AV200) or low (AV100) dose resulted in a considerable decline in cardiac serum levels of CK-MB, and LDH. Noticeably, the higher dose of AV dramatically altered the CK-MB level compared to the low dose AV supplementation (Figure 3).

### 3.3 Cardiac lipid peroxidation and antioxidant markers assay

**FIGURE 4:**
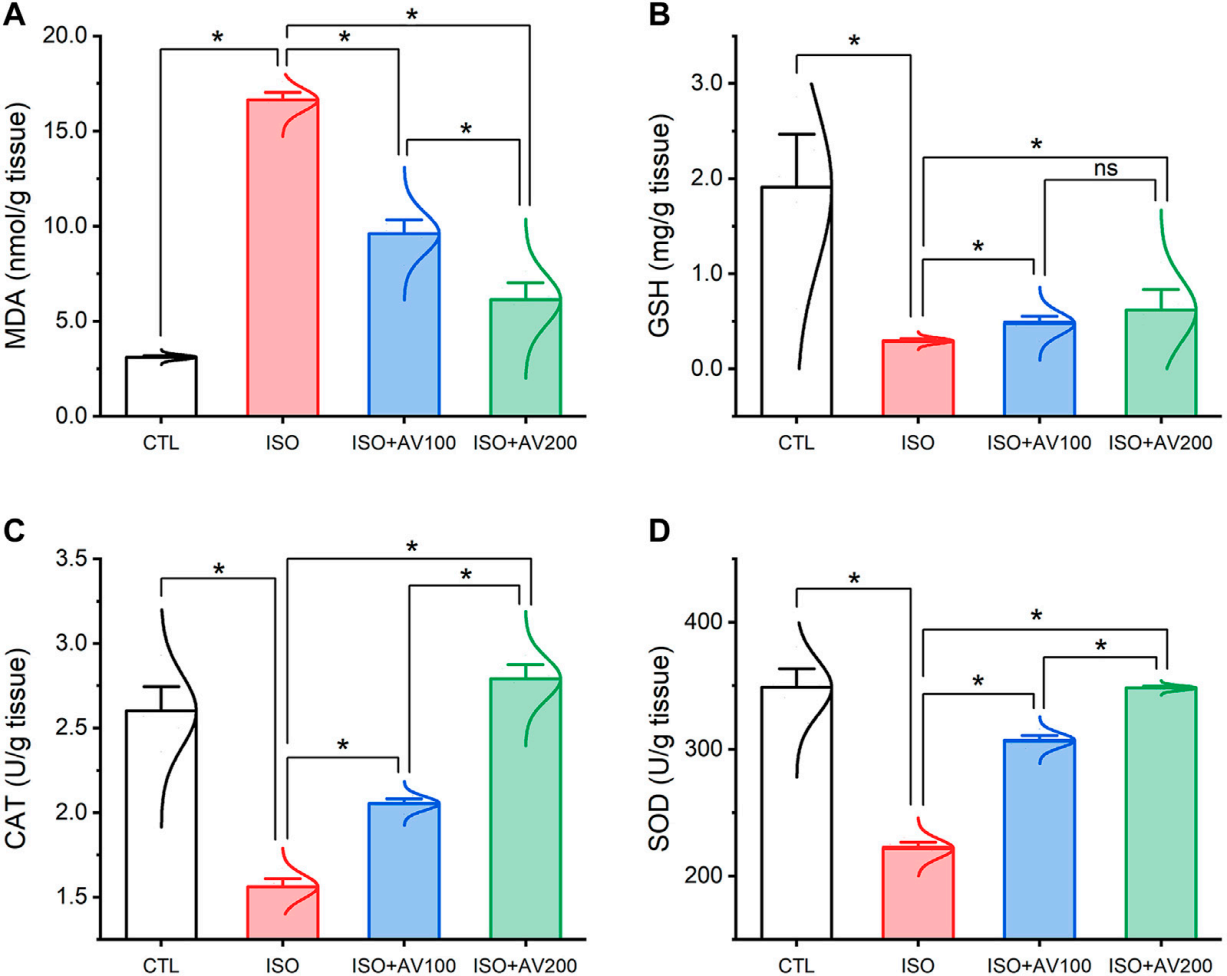
Bar plots of oxidative stress markers in cardiac tissue of ISO-exposed mice with AV supplementation. **(A)** MDA, malondialdehyde; **(B)** GSH, reduced glutathione; **(C)** CAT, catalase; **(D)** SOD, superoxide dismutase. Values are expressed as means ± SEM (*p < 0.05). Colored spikes indicate the data distribution. AV, *Alchemilla vulgaris*; CTL, control; and ISO, isoproterenol.

ISO triggered substantial oxidative injury and lipid peroxidation (LPO), as depicted in Figure 4. A remarkable increase in the cardiac MDA levels accompanied by a discernible decrease in the enzyme activities of SOD and CAT and the GSH level was observed in mice treated with ISO in contrast to the other groups. Notably, the ISO-prompted oxidative stress was considerably reduced by AV supplementation, as indicated by a substantial decrease in the MDA level and drastic increase in GSH, SOD, and CAT indices in a dose-dependent manner.

### 3.4 Inflammatory pathway

**FIGURE 5:**
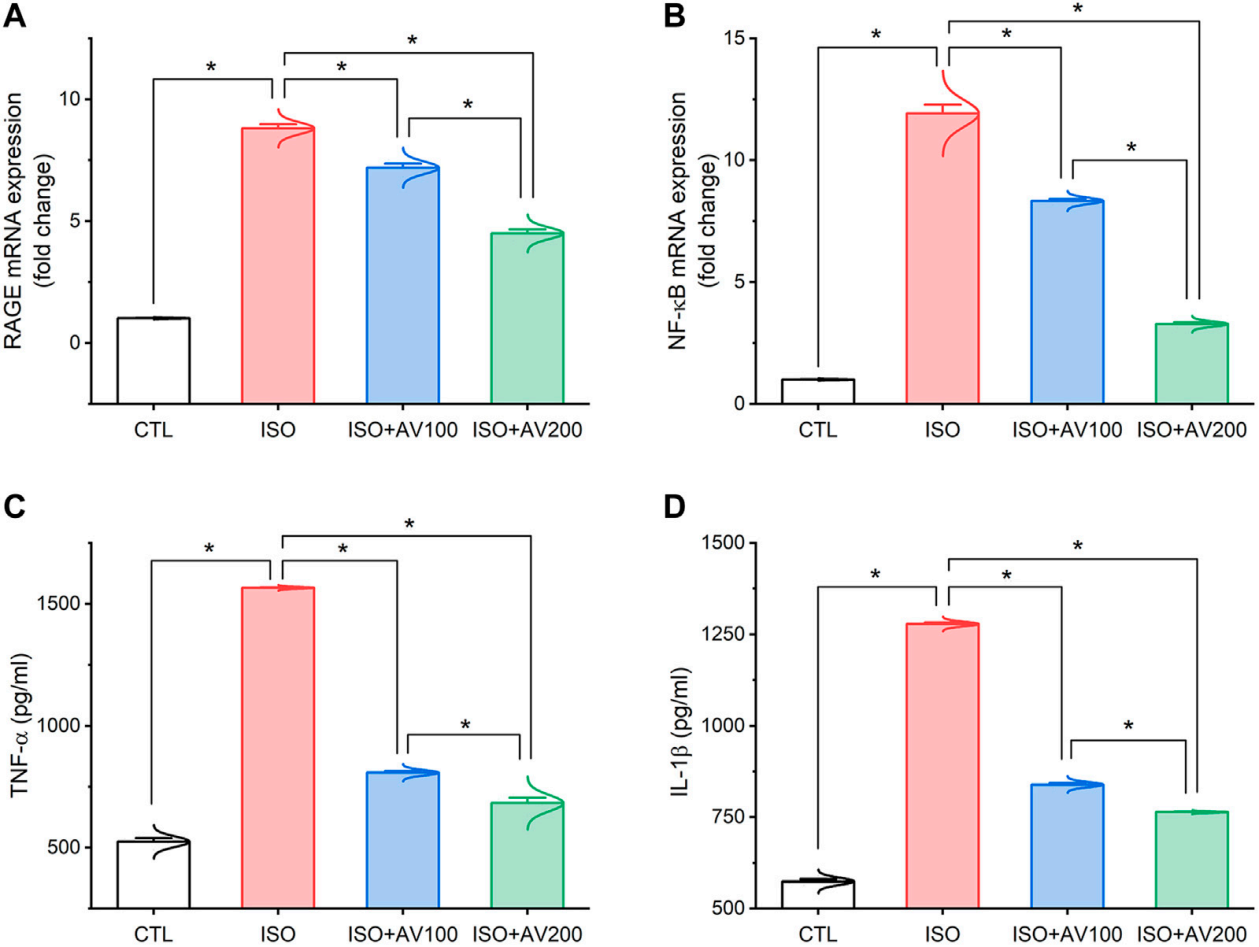
Bar plots of inflammation-related genes and inflammatory cytokines in cardiac tissue of ISO-exposed mice with AV supplementation. **(A)** mRNA expression of RAGE, receptor for advanced glycation end products; **(B)** mRNA expression of NF-κB/p65, nuclear factor kappa-B transcription factor/p65; **(C)** TNF-α, tumor necrosis factor alpha; **(D)** IL-1β, interleukin-1β. Values are expressed as means ± SEM (*p < 0.05). Colored spikes indicate the data distribution. AV, *Alchemilla vulgaris*; CTL, control; and ISO, isoproterenol.

As depicted in Figure 5, the ISO-exposed mice exhibited cardiac inflammation. This was elucidated by a dramatic increase in the levels of pro-inflammatory cytokines (TNF-α, and IL-1β) along with upregulation of NF-κB/P65 mRNA levels and RAGE inflammation-related genes in the ISO- intoxicated group in comparison to the other groups. Remarkably, pretreatment with AV100 and AV200 showed considerable decreases in ISO-induced cardiac inflammation in a dose-dependent pattern.

### 3.5 Autophagy and apoptotic pathways

**FIGURE 6:**
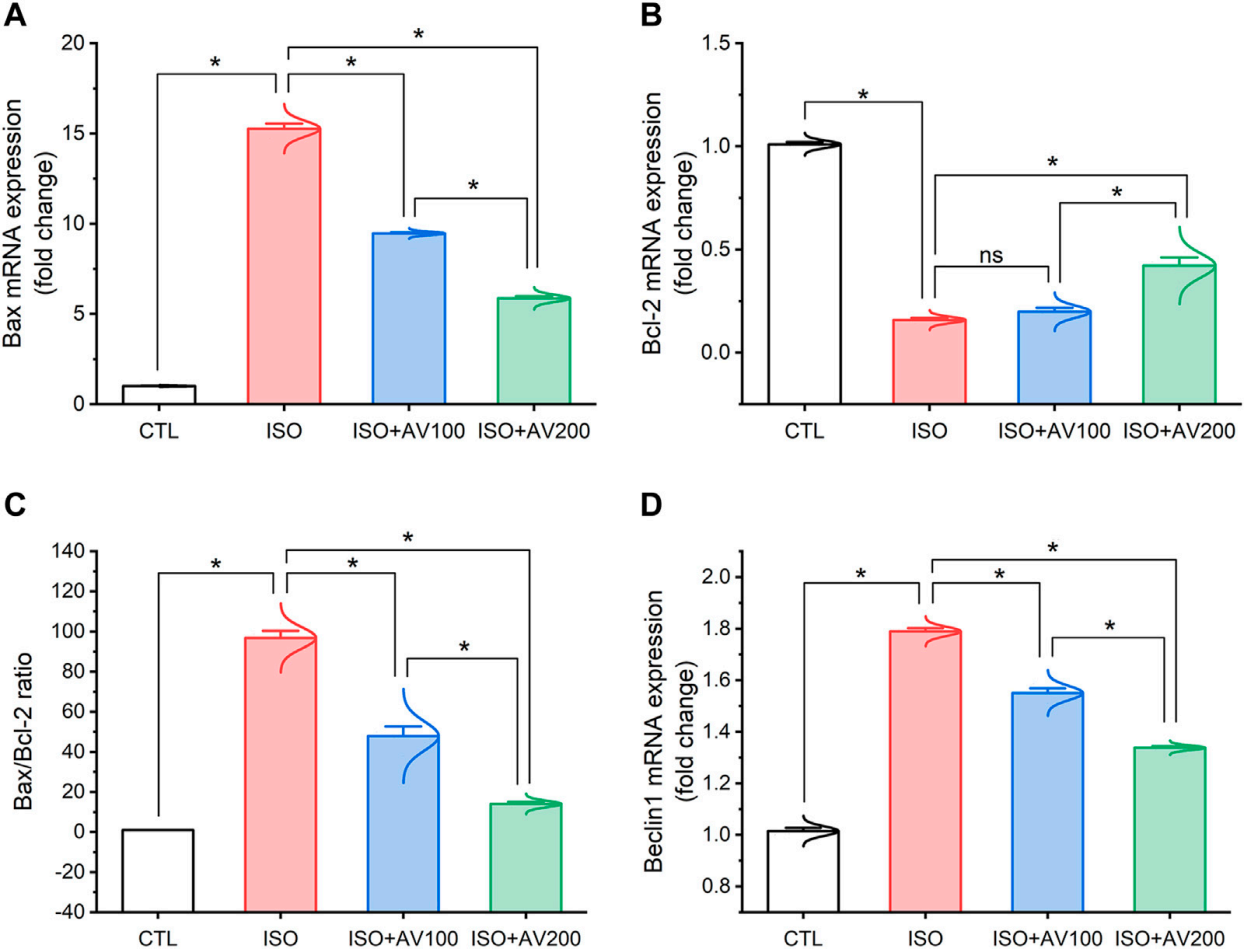
Bar plots of apoptosis and autophagy-related genes in cardiac tissue of ISO-exposed mice with AV supplementation. **(A)** mRNA expression of Bax, **(B)** mRNA expression of Bcl-2, **(C)** Bax/Bcl-2 ratio, **(D)** mRNA expression of Beclin1. Values are expressed as means ± SEM (*p < 0.05). Colored spikes indicate the data distribution. AV, *Alchemilla vulgaris*; CTL, control; and ISO, isoproterenol.

ISO exposure induced cardiac apoptosis as indicated by alterations in mRNA expression of apoptotic and anti-apoptotic biomarkers (Figure 6). Compared to the control mice, ISO exposure substantially increased the expression of Bax and induced considerable downregulation of Bcl-2 (anti-apoptotic) gene expression levels, indicating stimulation of apoptotic cell death. On the other hand, we observed well-regulated expression of these genes in cardiac tissue when ISO-exposed mice were pretreated with AV (low or high doses) in a dose-dependent pattern. Together, these results indicated the promotion of the apoptotic pathway with ISO exposure. Furthermore, ISO exposure induced cardiac autophagy as exhibited by upregulation of Beclin1 mRNA expression compared to control mice. In addition, AV treatment significantly modulated Beclin1 expression in a dose-response manner.

### 3.6 HMGB1 and LC3 expression in the cardiac tissue

**FIGURE 7:**
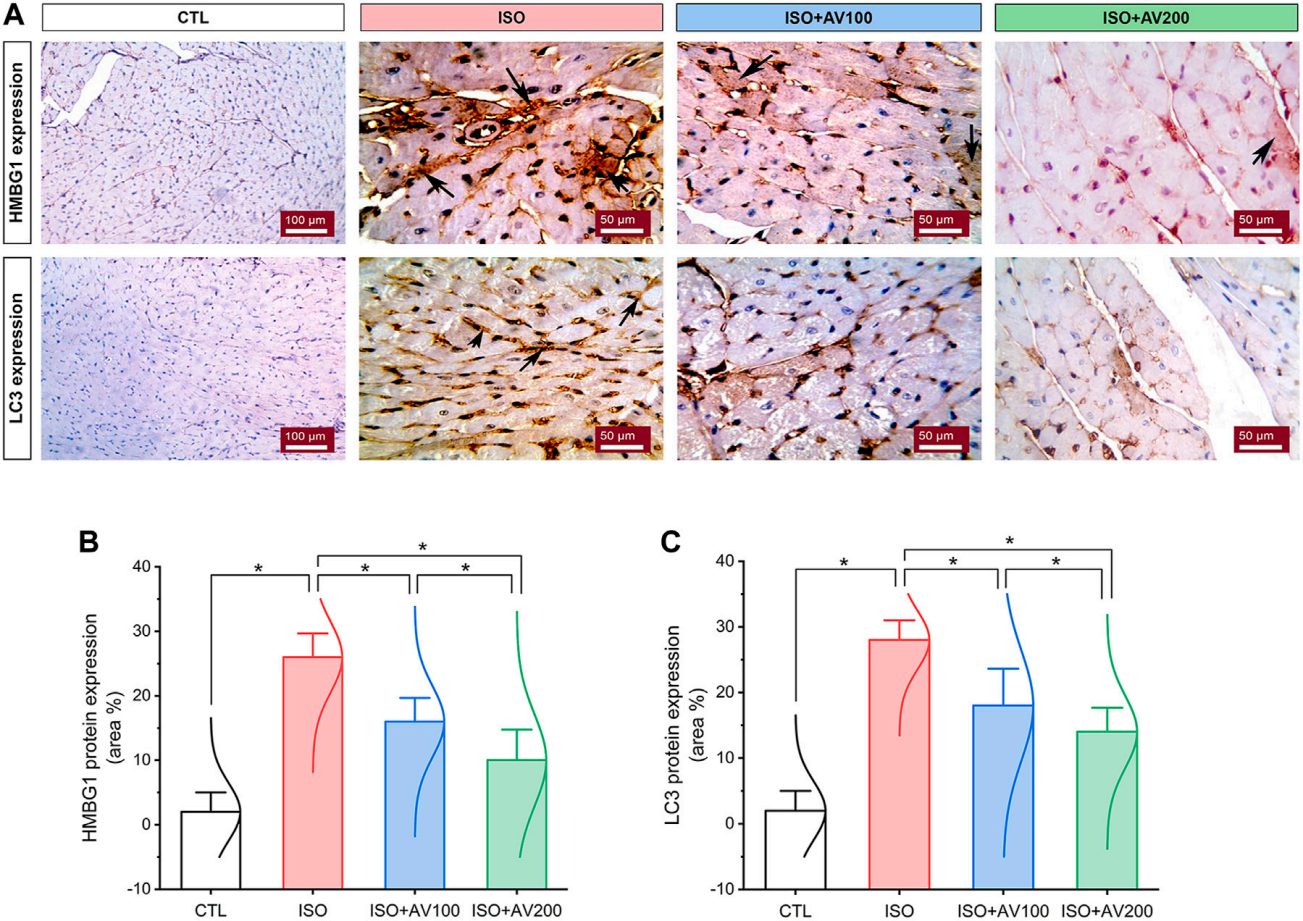
Changes in HMBG1 and LC3 expression in cardiac sections of ISO-exposed mice with AV supplementation. **(A)** CTL group exhibits passive expression of both proteins. Prominent positive brown staining of cardiomyocytes in the ISO group. ISO + AV100 and ISO + AV200 groups exhibited moderate and minimal expression of both proteins, respectively. The positive staining is indicated by the arrows and the brown color. **(B)** Quantitative analysis of protein expression of HMBG1. **(C)** Quantitative analysis of protein expression of LC3. Values are expressed as means ± SEM (*p < 0.05). Colored spikes indicate the data distribution. AV, *Alchemilla vulgaris*; CTL, control; and ISO, isoproterenol.

HMBG1 and LC3 expression in cardiac tissue sections of ISO-exposed mice with AV supplementation are shown in Figure 7. ISO exposure resulted in considerable upregulation of HMBG1 and LC3 expression in cardiac tissue (prominent positive brown staining of cardiomyocytes). In contrast, we observed moderate to minimal expression of both proteins when mice were pre-treated with AV100 and AV200, respectively. The semiquantitative analysis of positive-stained sections in the different treatment groups corroborated these results. Therefore, the results revealed improved in ISO-upregulated HMBG1 and LC3 expression following AV pretreatment. Intriguingly, the high dose of AV (200 mg/kg) reduced the ISO-mediated cardiac injury substantially and in a dose-dependent manner.

### 3.7 Cardiac histoarchitecture

**FIGURE 8:**
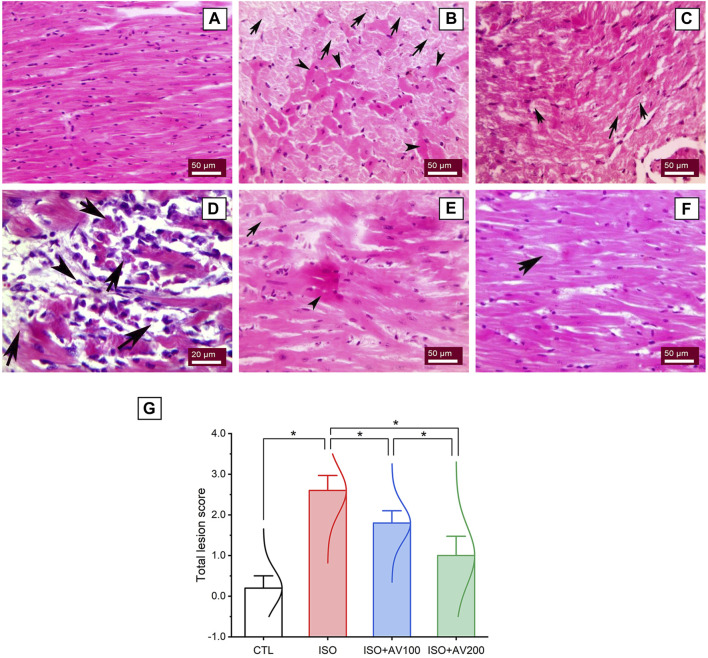
Histoarchitectural changes in cardiac tissue of ISO-exposed mice with AV supplementation. **(A)** Control group exhibits normal cardiac structure. **(B, C)**, ISO-treated group shows Zenker’s degeneration (arrowheads) and necrotic changes (arrows) in the myocardial fibers and degranulation in the cardiomyocytes. **(D)** leukocytic infiltrations (arrowheads) replaced the necrotic myocardial muscle fibers (arrows). **(E)** ISO + AV100 group presents mild degenerative changes in almost all cardiac tissue sections, with little vacuolation. **(F)** ISO + AV200 group depicts a remarkable recovery of cardiac histology. **(G)** Lesion scores of histopathological alterations of cardiac cells among the different treatment groups. Values are expressed as means ± SEM (*p < 0.05). Colored spikes indicate the data distribution. AV, *Alchemilla vulgaris*; CTL, control; and ISO, isoproterenol.

A histological assessment was done to confirm the previously reported findings that evaluated alterations in cardiac tissue sections in ISO-exposed mice that received AV supplementation. The control mice showed normal cardiac tissue architecture, including normal arrangement of muscle fibers, normal elongated branching pattern, and oval central striated nuclei, as shown in Figure 8A. On the other hand, mice exposed to ISO (Figures 8B–D) exhibited overt cardiac damage, as indicated by various degenerative and necrotic changes in myocardial fibers and cardiomyocytes. Degranulation, vacuolation of the cytoplasm, and leukocytic cellular infiltration also were observed. Nevertheless, pre-treatment of ISO by AV100 demonstrated moderate improvement in the histological findings, as indicated by mild degenerative changes in nearly all the cardiac tissue sections, with little vacuolation (Figure 8E). Interestingly, myocardial histological architecture was nearly completely recovered following the high AV dose (absence of myocardial degenerative changes) (Figure 8F). The histopathological findings corroborated the biochemical data, indicating that pre-exposure to AV exhibited a profound protective impact against ISO-inflicted cardiac damage in a dose-dependent manner (Figure 8G).

### 3.8 Multivariate analyses

**FIGURE 9:**
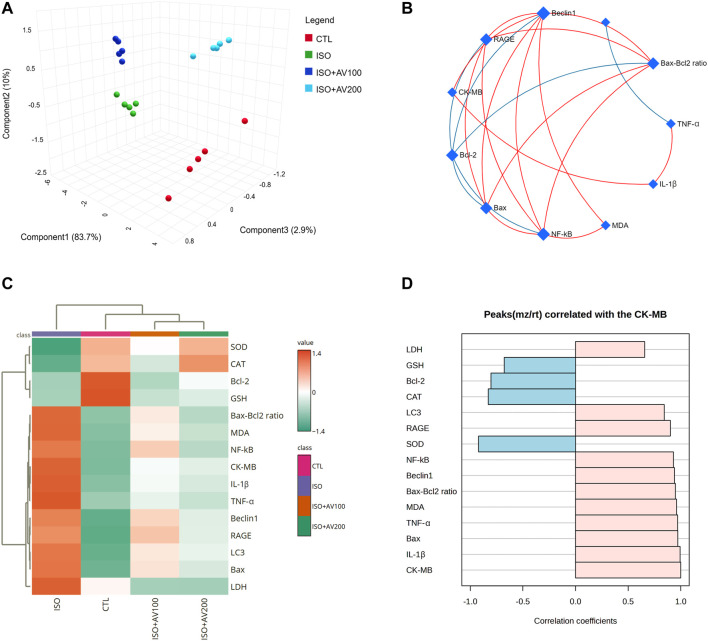
Clustering analysis of whole datasets in cardiac tissue of ISO-exposed mice with AV supplementation using Pearson’s rank correlation coefficient. **(A)** 3D plot of principal component analysis (PCA) identifying the four experimental groups (CTL, ISO, ISO + AV100, and ISO + AV200). **(B)** Correlation network. The measured variables are represented by the nodes in the network, while the correlation measures are represented by the edges. Line color is proportional to the strength of the correlation. The blue lines display a negative correlation, while the red lines display a positive correlation. The node size is proportional to the mean abundance of measured variables. **(C)** Clustering heatmap. Each colored cell in the map represents a concentration value, and the rows and columns are made of different averages and treatment sets, respectively. Dark orange has the highest value on the gradation scale, while green has the lowest. **(D)** A pattern hunter plot correlation coefficient between CK-MB level in cardiac muscle and other measured parameters. Pink bars represent the positive correlation (Pearson r > +0.5) while blue bars show the negative correlation (Pearson r > −0.5).

Multivariate analyses were performed to ascertain the correlation between the measured parameters and treatment groups, as depicted in Figure 9. Principal component analysis (PCA) was used to investigate the association between different treatments and covariates (Figure 9A). The PCA distinguished three main dimensional components for all variables, which collectively represented 96.6% of the variation. Component 1 discriminated most of the examined variables and therefore expressed the larger proportion of variation (83.7%), whereas components 2 and 3 accounted for smaller proportions of the variance. The PCA revealed that the ISO-exposed group was clearly different from the control group and the ISO + AV200 group. These findings elucidated a significant distinction between the AV pretreated animals and those exposed to ISO.

In addition, correlation networks were constructed among variables in the control and exposed groups (Figure 9B). Beclin1 and NF-κB/p65 correlated positively with each other and with RAGE, Bax, Bax-Bcl2 ratio, and MDA. Beclin1 and NF-κB/p65 were negatively correlated with Bcl-2. In addition, CK-MB was positively correlated with RAGE, Beclin1, and IL-1β. Furthermore, the clustering heatmap provided an intuitive visualization of all data sets (Figure 9C). The clustering heatmap revealed a noticeable distinction between the concentration values of all evaluated variables following ISO exposure in contrast to the other treatment groups. These data demonstrated that the ISO-exposed mice displayed greater injury than mice in the other groups. On the other hand, the intensity of the cell color with AV treatment revealed an intermediate color density at all measured variables, indicating the mitigating effect of AV against ISO exposure.

Finally, we performed a correlation analysis to investigate the relationship of CK-MB levels with other measured parameters. Figure 9D depicts the different parameters positively or negatively correlated with the CK-MB level in cardiac tissue. All measured variables had a strong positive correlation with CK-MB level, while only GSH, SOD, CAT, and Bcl-2 exhibited negative correlation.

## 4 Discussion

The current *in silico* study explored whether ISO had a binding affinity to glutathione synthetase, CAT, SOD1, SOD2, SOD3, and Bcl-2, which revealed the association of oxidative and apoptotic pathways to ISO-induced cardiac injury. In addition, AV’s bioactive compounds exhibited binding affinity to TNF-α, IL-1β, NF-κB1, Bax, RAGE, and HMBG1, which attests to potential antioxidant, anti-inflammatory, and antiapoptotic activities.

ISO is categorized as a synthetic, non-selective, β-adrenergic agonist (Pandi et al., 2022). It produces significant cardiotoxicity, particularly infarct-like damage in the myocardium. Several mechanisms have been proposed in the pathogenesis of ISO-induced myocardial damage, but the production of ROS during autoxidation of catecholamines is one of the most critical causal reasons (Song et al., 2020). Quinone is produced when ISO is oxidized, producing a range of free radicals that cause LPO and exhaustion of the cellular antioxidant system (Othman et al., 2017; Song et al., 2020). This study provided compelling evidence that ROS contributed to the toxic consequences of ISO treatment, which is exhibited by noteworthy reductions in GSH levels and enzymatic activities of SOD and CAT in cardiac tissues. SOD is the first line of enzymatic defense in mitochondria and responsible for the dismutation of the generated O_2_^•−^ to O_2_ and H_2_O_2_ (Abdeen et al., 2023;Shanab et al., 2024) Furthermore, CAT accelerates the dissolution of H_2_O_2_ into water and O_2_, quenching oxidative damage (Hassan et al., 2023). In the case of CAT exhaustion, substantial quantities of OH^•^, the most potent reactive radical, are generated from H_2_O_2_ by Fenton’s reaction. The OH^•^ molecules drastically destroy lipid membranes, producing LPO and boosting the production of another harmful substance, MDA (Abdelnaby et al., 2022). MDA itself can dramatically disrupt the membrane potential of mitochondria and alter cellular proteins and DNA integrity, leading to substantial cellular damage (Shanab et al., 2023). Our investigation documented substantial increase in MDA levels, corroborating the possibility of membrane damage due to ISO exposure.

As anticipated, the enhanced LPO disrupted the membrane integrity and permeability of the cardiomyocytes, liberating cardiac enzymes (CK-MB and LDH) into the blood, elevating their levels (Liu et al., 2018). This result was confirmed by our correlation analysis, which revealed a robust positive correlation between CK-MB levels and MDA accumulation in cardiac muscle. Our oxidative stress markers were consistent with earlier research that reported a reduction in cellular antioxidants upon exposure to ISO (Abdel-hady et al., 2021). Furthermore, the result mentioned above correlated with those of Sammeturi et al. (2019), who corroborated LPO and enhanced MDA levels following ISO treatment. The enhanced LPO corresponded with our histology findings, which revealed degenerative alterations within the cardiomyocytes and vividly mirrored the biochemical findings.

Mounting evidence revealed a robust correlation between inflammation and oxidative stress. Therefore, we suggest that the inflammatory pathway is an additional mechanism underlying ISO-induced cardiotoxicity (Garg and Khanna, 2014; Jain et al., 2018). Increased ROS production triggers a cellular signaling cascade that enhances the expression of proinflammatory genes and releases inflammatory mediators, culminating in enhanced inflammation (Shanab et al., 2023). In accordance with the outcomes of this study, ISO considerably increases cardiac levels of proinflammatory cytokines (IL-1β, and TNF-α) along with enhanced HMGB1 immunostaining. Moreover, the expression of NF-κB/p65 and RAGE m-RNA genes were upregulated. The involvement of the HMGB1–RAGE axis in the pathogenesis of inflammatory cardiac disease has received considerable attention recently (Bangert et al., 2016). HMGB1 binds to RAGE and Toll-like receptors in response to cellular injury, causing the release of chemokines and inflammatory cytokines (Bangert et al., 2016; Habotta et al., 2023). RAGE is a cell membrane receptor expressed in cardiomyocytes (Zhang et al., 2021; Dong et al., 2022), during cell death, it can be passively released and actively secreted into the extracellular space (Yang et al., 2023).

RAGE signaling also can provoke an inflammatory reaction via amplifying various cellular cascades that enhance inflammatory cytokine release and promote activation of the NF-κB/p65 pathway, culminating in an enormous inflammatory reaction (Jangdea et al., 2020; Habotta et al., 2023). NF-κB/p65 is a redox-regulated transcription factor that is inactive under normal conditions, but when triggered, enters the nucleus (Habotta et al., 2023; Hassan et al., 2023) and promotes proinflammatory mediators, including TNF-α that enhance the activation of IL-6 and adhesion molecules, encouraging leukocytes to congregate at the zone of inflammation (Shanab et al., 2024). Our correlation network further confirmed the robust relationship between oxidative stress and inflammation with ISO-induced cardiac damage. Furthermore, our histological evaluation of heart tissue verified these effects, as indicated by notable infiltration of inflammatory cells. These findings are consistent with previous studies that reported enhanced expression of these inflammation-associated markers in the cardiac tissue (Liu et al., 2018; Asiwe et al., 2023). Besides, Bangert and his gorup observed considerable increases in the expression of HMGB1 and RAGE, in myocardial biopsies of patients suffering from acute myocarditis (Bangert et al., 2016).

It has been documented that mitochondrial oxidative stress triggers numerous cell signaling pathways, including apoptosis (Meeran et al., 2021). Apoptosis is cell death brought on by the initiation of specific cellular programs controlled by complex regulatory mechanisms (Xiao et al., 2019; Sun et al., 2024). However, autophagy, a lysosomal breakdown pathway, is responsible for the degradation of dysfunctional and superfluous organelles and proteins which plays a crucial role in cell survival and homeostasis (El-Ashmawy et al., 2022; Sun et al., 2024). The relationship between apoptosis and autophagy is complicated, as the balance between autophagy and apoptosis is essential for cell survival (Wang et al., 2018). Apoptosis is regulated by two cytoplasmic proteins, pro-apoptotic Bax and anti-apoptotic Bcl-2. In apoptosis, Bax protein triggers the cascade of mitochondrial intrinsic pathways, eventually leading to cell death (Kulsoom et al., 2018). On the other hand, Bcl-2 prevents apoptosis by suppressing the Bax proteins, restricting downstream activation of apoptotic machinery pathways (Kulsoom et al., 2018; Sun et al., 2024). In line with this evidence, our investigation discovered enhanced apoptosis as shown by marked increased mRNA expression levels of Bax alongside downregulation of Bcl-2 in myocardial tissue of the ISO-treated group in contrast to the control group.

On the other hand, Beclin1 is the critical gene that regulates cardiac autophagy and autophagosome formation (Yang et al., 2023). Bcl-2 binds to Beclin1 forming a Beclin1/Bcl-2 complex. Consequently, Beclin1 is sequestered, and autophagy is suppressed (Dong et al., 2019). HMGB1, a critical modulator of autophagy, is translocated from the nucleus to the cytosol via a ROS-dependent pathway (Tang et al., 2010). Then, it binds to Beclin1 and encourages its dissociation from Bcl-2, thereby enhancing the autophagic flux (Dong et al., 2019; Yang et al., 2023). In addition, HMGB1 is passively secreted into the extracellular space following cell death and modulates cellular autophagic activity by binding with RAGE (Yang et al., 2023). Moreover, LC3 is a well-known biomarker of autophagy activity that strongly correlates with autophagosome biogenesis (Xiao et al., 2019). Our investigation documented autophagy dysfunction, as indicated by upregulation of Beclin1 m-RNA expression alongside exaggerated immunostaining for LC3 and HMGB1 proteins in myocytes following ISO exposure. In accordance with our data, El-Ashmawy et al. (2022) and Sun et al. (2024) reported myocardial autophagy following ISO treatment that was expounded by overexpression of the initiation marker LC3.

AV is extensively used in folk remedies throughout the world due to its well-known anti-inflammatory and immunomodulatory effects (Boroja et al., 2018; Kanak et al., 2022), as well as its antioxidant capabilities (Jain et al., 2021; Jelača et al., 2022). AV’s antioxidant activity is the primary plausible mechanism underlying its protective action against heart injury (Yücel and Yücel, 2020; Jakimiuk and Tomczyk, 2024). This study also revealed that AV treatment could have a robust antioxidant impact on the oxidative stress induced by ISO in a dose-dependent manner. The protective capability of AV to scavenge free radicals is attributed to its richness in total polyphenolic and flavonoid content, especially the very high concentrations of soluble tannins and saponins (El-Hadidy et al., 2018; Jurić et al., 2020; Jakimiuk and Tomczyk, 2024). The hydroxyl groups found in the chemical structure of phenolic compounds might offer critical reasons for their ability to function as radical scavengers (Jain et al., 2021). In addition, quercetin, one of the phytochemicals in AV, can decrease the risk of arteriosclerosis by inhibiting the oxidation of low-density lipoprotein, which reduces cardiac damage (Yücel and Yücel, 2020). Many compelling publications have endorsed the idea that AV supplementation boosts the effectiveness of antioxidant enzymes and mitigates the action of LPO (Jurić et al., 2020; Tadić et al., 2020).

Notably, ongoing research indicates that pre-treatment with AV could alleviate the cardiac damage triggered by ISO, as revealed by the considerable improvement in cardiac serum biomarkers in a dose-dependent manner. The abundance of heart-strengthening compounds in AV such as pedunculin, agrimoniin, sanguin, castalagin, vescalagin, gallic, and chlorogenic acids may have critical roles in these actions (Yücel and Yücel, 2020). Furthermore, these flavonoids have been reported to have cardioprotective effects (Said et al., 2010).

**FIGURE 10:**
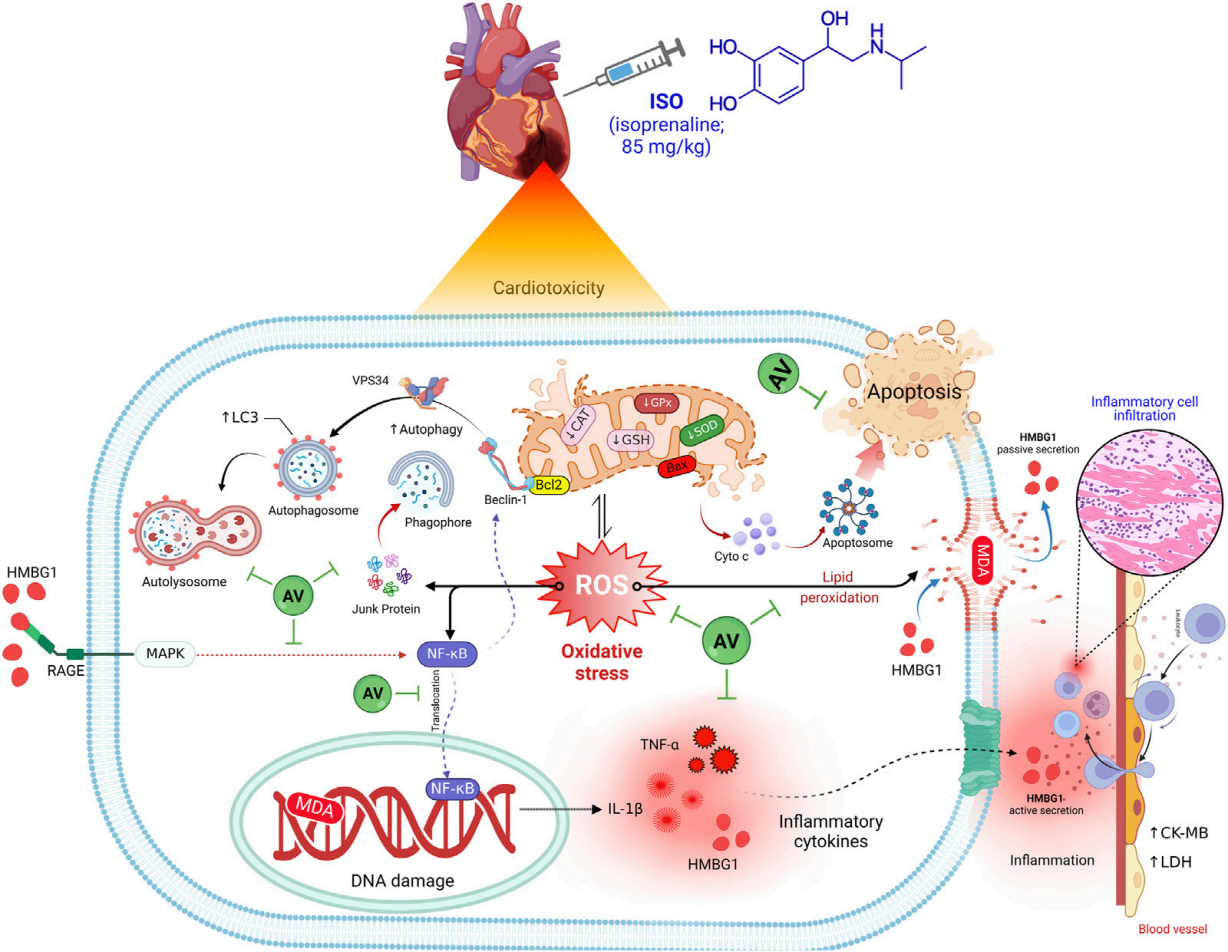
The molecular pathways behind AV’s mitigating function following ISO exposure.

The current work also revealed a dose-dependent, immune-modulating action of AV in ISO-exposed mice, which was elucidated by alterations in the expression levels of proinflammatory cytokines and suppression of inflammatory cell infiltration into cardiac tissues. Intriguingly, AV is a rich source of ellagitannins that have a crucial function in alleviating the inflammatory response via the NF-κB/p65 pathway, thus, inhibiting the release of proinflammatory cytokines (Schink et al., 2018). Our findings align with other previous reports (Katare et al., 2017; Jelača et al., 2022). Also, AV supplementation exerted an anti-apoptotic effect against ISO-prompted enhanced mRNA expression of Bax and downregulated Bcl-2 mRNA expression in cardiomyocytes. Additionally, both doses of AV downregulated the expression of Beclin1 and LC3 proteins and affected cardiac autophagy, which is a potential mitigating mechanism against ISO-triggered myocardial damage. Figure 10 highlights the molecular pathways underlying AV’s mitigating function following ISO exposure.

## 5 Conclusion

Our data elucidated the potential mitigating effect of AV on ISO-inflicted cardiac injury. This effect was likely due to AV’s enriched antioxidant, ROS-scavenging, and anti-inflammatory attributes. Mice pretreated with AV maintained the integrity of cardiac tissue architecture and function. AV exerted notable improvements in the heart’s antioxidant defenses by reducing the levels of MDA and increasing GSH levels and SOD and CAT activities. Notable decreases in the gene expression of pro-inflammatory cytokines and NF-κB/p65 were observed in the AV-treated mice. Targeting the HMGB1/RAGE signaling pathway also contributed to the cardioprotective action of AV. Furthermore, AV alleviated the ISO-induced apoptotic death and autophagy. Thus, our data suggest that AV supplementation is a promising treatment for ISO-induced cardiotoxicity that acts in a dose-dependent manner.

## Data Availability

The original contributions presented in the study are included in the article/Supplementary Material, further inquiries can be directed to the corresponding authors.

## Abbreviations

AV, *Alchemilla vulgaris*; Bcl-2, B-cell lymphoma protein 2; Bax, Bcl-2-associated X; CAT, catalase; CK-MB, creatine kinase-myoglobin binding; GSH, reduced-glutathione; HMGB1, high mobility group box 1 protein; ISO, isoproterenol; IL-1β, interleukin-1β; LDH, lactate dehydrogenase; LPO, lipid peroxidation; MDA, malondialdehyde; LC3, microtubule‑associated protein light chain 3; NF-κB/p65, nuclear factor Kappa-B/p65; PCA, principal component analysis; RAGE, receptor of advanced glycation end products; SOD, superoxide dismutase; TNF-α, tumor necrosis factor-α.
