# Correction: *Alchemilla vulgaris* modulates isoproterenol-induced cardiotoxicity: interplay of oxidative stress, inflammation, autophagy, and apoptosis

**DOI:** 10.3389/fphar.2025.1650419

**Published:** 2025-08-13

**Authors:** Nuha Anajirih, Ahmed Abdeen, Ehab S. Taher, Afaf Abdelkader, Hoda A. Abd-Ellatieff, Mahmoud S. Gewaily, Nashwa E. Ahmed, Rasha H. Al-Serwi, Safwa M. Sorour, Heba M. Abdelkareem, Elturabi Ebrahim, Mohamed El-Sherbiny, Florin Imbrea, Ilinca Imbrea, Mahmoud M. Ramadan, Ola A. Habotta

**Affiliations:** ^1^ Department of Medical Emergency Services, College of Health Sciences in Al-Qunfudah, UmmAl-Qura University, Mecca, Saudi Arabia; ^2^ Department of Forensic Medicine and Toxicology, Faculty of Veterinary Medicine, Benha University, Toukh, Egypt; ^3^ Department of Basic Medical and Dental Sciences, Faculty of Dentistry, Zarqa University, Zarqa, Jordan; ^4^ Department of Forensic Medicine and Clinical Toxicology, Faculty of Medicine, Benha University, Benha, Egypt; ^5^ Department of Pathology, Faculty of Veterinary Medicine, Damanhour University, Damanhour, Egypt; ^6^ Department of Anatomy and Embryology, Faculty of Veterinary Medicine, Kafrelsheikh University, Kafrelsheikh, Egypt; ^7^ Department of Medical Biochemistry and Molecular Biology, Faculty of Medicine, Benha University, Benha, Egypt; ^8^ Department of Basic Dental Sciences, College of Dentistry, Princess Nourah bint Abdulrahman University, Riyadh, Saudi Arabia; ^9^ Department of Pharmacology, Faculty of Medicine, Benha University, Benha, Egypt; ^10^ Department of Medical Biochemistry, Molecular Biology and Physiology, Faculty of Medicine, Mutah University, Mutah, Jordan; ^11^ Medical-Surgical Nursing Department, College of Applied Medical Sciences, Prince Sattam bin Abdulaziz University, Al-Kharj, Saudi Arabia; ^12^ Department of Basic Medical Sciences, College of Medicine, AlMaarefa University, Riyadh, Saudi Arabia; ^13^ Department of Agricultural Technologies, Faculty of Agriculture, University of Life Sciences “King Mihai I” from Timisoara, Timisoara, Romania; ^14^ Department of Forestry, Faculty of Engineering and Applied Technologies, University of Life Sciences “King Mihai I” from Timisoara, Timisoara, Romania; ^15^ Department of Clinical Sciences, College of Medicine, University of Sharjah, Sharjah, United Arab Emirates; ^16^ Department of Cardiology, Faculty of Medicine, Mansoura University, Mansoura, Egypt; ^17^ Department of Forensic Medicine and Toxicology, Faculty of Veterinary Medicine, Mansoura University, Mansoura, Egypt

**Keywords:** Alchemilla vulgaris, isoproterenol, myocardial injury, oxidative stress, inflammatory cytokines, HMBG1/RAGE pathway

Supplemental material Supplementary File 1 was omitted. The file has now been published.

