# Supplementary material for: Correction: Alchemilla vulgaris modulates isoproterenol-induced cardiotoxicity: interplay of oxidative stress, inflammation, autophagy, and apoptosis
Source: Front Pharmacol. 2025 Aug 13;16:1650419. doi: 10.3389/fphar.2025.1650419 (PMC12381503; doi:10.3389/fphar.2025.1650419)
Supplement: Supplementary file 1 [file Supplementaryfile1.docx]

**Full taxonomy of Alchemilla vulgaris:**

- **Domain**: Eukaryota
- **Kingdom**: Plantae (Plants)
- **Phylum**: Tracheophyta (Vascular plants)
- **Class**: Magnoliopsida (Dicotyledons)
- **Order**: Rosales
- **Family**: Rosaceae (Rose family)
- **Genus**: Alchemilla (Lady's Mantle)
- **Species**: Alchemilla vulgaris L.

**Methods of HPLC analysis of *Alchemilla vulgaris* extract**

The analysis was carried out by HPLC-(Agilent 1100) with two LC-pumps, a UV/Vis detector, and a C18 column (125 mm × 4.60 mm, 5 µm particle size). Chromatograms were obtained and analyzed using the Agilent Chem Station. The mobile phase was treated using ultrasounds to remove the dissolved air before being filtrated via a PTFE 0.2 m membrane. Phenolic acids were isolated by utilizing a gradient mobile phase of two solvents: solvent A; Methanol and solvent B; acetic acid in water 1:25, at wavelength of 250 nm. The mobile phase employed for the separation of flavonoids was acetonitrile (A) and 0.2% (v/v) aqueous formic acid (B) with an Isocratic elution (70:30) program at 360 nm wavelength. The graded program began with 100% B and was held at this concentration for the 1^st^ 3 min. This was followed by 50% solvent A for the next 5 min, after which the concentration of A was raised to 80% for the next 2 min and then lowered to 50% again for the following 5 min. The identified compounds were compared their retention times and absorption spectra of the peaks with reference samples, and their concentrations were determined from calibration curves to their standard compounds. All standards (catechol, syringenic acid, caffeic acid, benzoic acid, gallic acid, cinnamic acid, ellagic acid, chrysoeriol, quercetin, kaempferol, luteolin, hesperidin, catechin) were purchased from Sigma-Aldrich (St. Louis, MO, USA).

**Table S1.** Phenolic and flavonoid compounds in AV extract

| **RT** | **Compound** | **Concentration (μg/ml)** |
| --- | --- | --- |
| **I. Phenolics** | | |
| 4.1 | Catechol | 5.33 |
| 5.1 | syringenic | 7.12 |
| 7.0 | Benzoic | 2.19 |
| 8.0 | Caffeic | 1.98 |
| 9.8 | Gallic | 2.09 |
| 12.8 | Cinnamic | 6.49 |
| 15.7 | Ellagic | 8.11 |
| **II.**  **Flavonoids** | | |
| 7.0 | Querstin | 12.39 |
| 8.0 | Kampferol | 1.56 |
| 9.0 | Luteolin | 13.7 |
| 10.0 | Hisperdin | 2.89 |
| 12.0 | Catechin | 15.41 |
| 15.0 | Chrysoeriol | 3.24 |


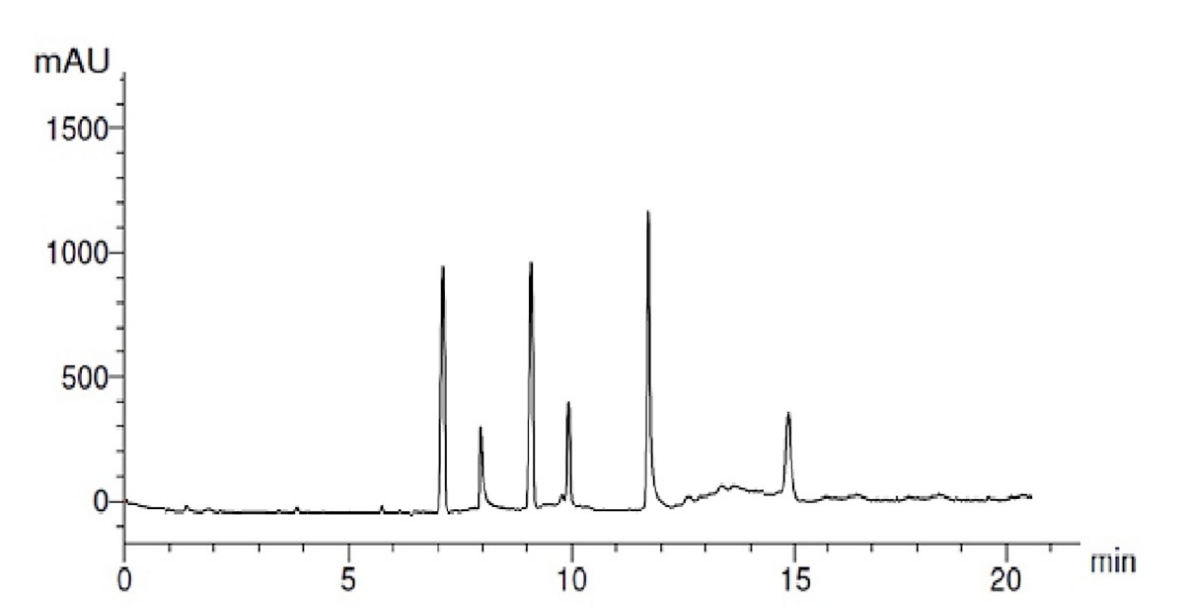


Flavonoids chromatogram


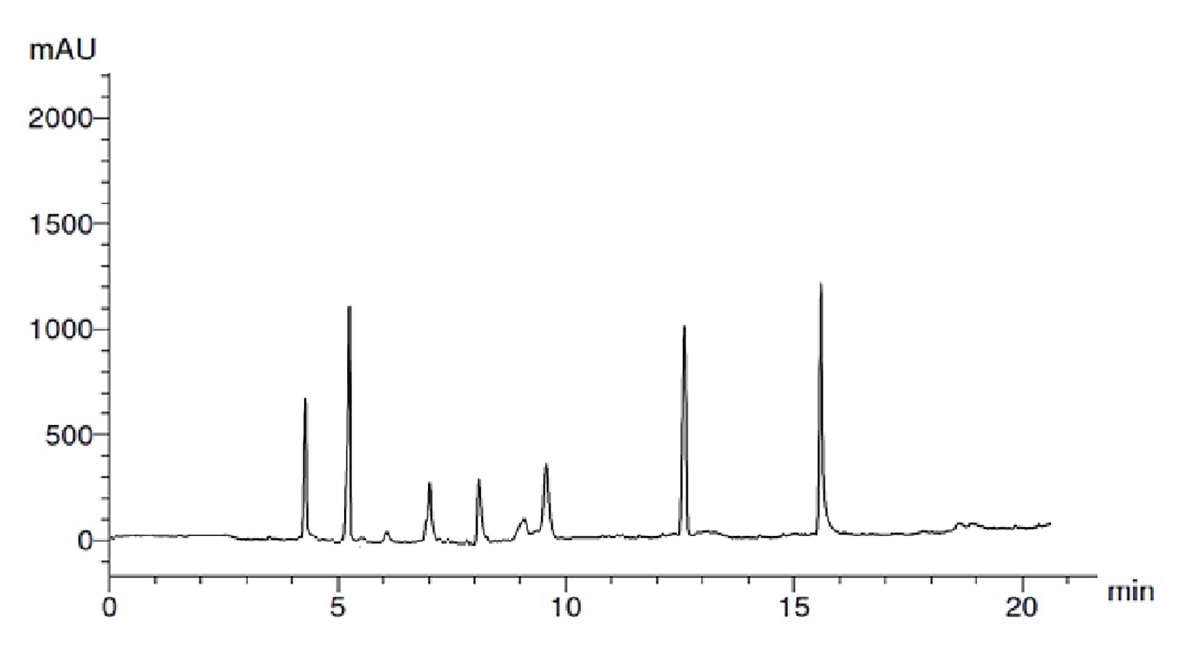


Phenolics chromatogram

**Table S2.** The primer sequences

| **Target gene** | **Primer sequences** | **Reference** |
| --- | --- | --- |
| *β-actin* | F: CCCCACTCCTAAGAGGAGGAT | (El-Khadragy et al., 2021) |
|  | R: CTCAGACCTGGGCCATTCAG |  |
| *Bcl-2* | F: GAGACTCACCAGGGTCTGCT | (El-Khadragy et al., 2021) |
|  | R: TACAGGGTGTGATAATGCCCCA |  |
| *Bax* | F: CACTGCCTTGGACTGTGTCT | (El-Khadragy et al., 2021) |
|  | R: CCTTTCCCCTTCCCCCATTC |  |
| *Beclin1* | F: GACGAACTCAAGAGTGTGGAGAACC | (He et al., 2022) |
|  | R: AGATGTGGAAGGTGGCATTGAAGAC |  |
| *RAGE* | F: ACTACCGAGTCCGAGTCTACC | (Shu et al., 2024) |
|  | R: CCCACCTTATTAGGGACACTGG |  |
| *NF-κB* | F: GAAATTCCTGATCCAGACAAAAAC | (Abdelhakm et al., 2023) |
|  | R: ATCACTTCAATGGCCTCTGTGTAG |  |
